# Private finance of services covered by the National Health Insurance package of benefits in Israel

**DOI:** 10.1186/s13584-015-0042-7

**Published:** 2015-12-28

**Authors:** Esti Engelchin-Nissan, Amir Shmueli

**Affiliations:** The Israeli Academic College, Ramat-Gan, Israel; The Hebrew University-Hadassah School of Public Health, Jerusalem, 91120 Israel

## Abstract

**Background:**

Private health expenditure in systems of national health insurance has raised concern in many countries. The concern is mainly about the accessibility of care to the poor and the sick, and inequality in use and in health. The concern thus refers specifically to the care financed privately rather than to private health expenditure as defined in the national health accounts.

**Objectives:**

To estimate the share of private finance in total use of services covered by the national package of benefits. and to relate the private finance of use to the income and health of the users.

**Methods:**

The Central Bureau of Statistics linked the 2009 Health Survey and the 2010 Incomes Survey. Twenty-four thousand five hundred ninety-five individuals in 7175 households were included in the data. Lacking data on the share of private finance in total cost of care delivered, we calculated instead the share of uses having any private finance—beyond copayments—in total uses, in primary, secondary, paramedical and total care. The probability of any private finance in each type of care is then related, using random effect logistic regression, to income and health state.

**Results:**

Fifteen percent of all uses of care covered by the national package of benefits had any private finance. This rate ranges from 10 % in primary care, 16 % in secondary care and 31 % in paramedical care. Twelve percent of all uses of physicians’ services had any private finance, ranging from 10 % in family physicians to 20 % in pulmonologists, psychiatrists, neurologists and urologists. Controlling for health state, richer individuals are more likely to have any private finance in all types of care. Controlling for income, sick individuals (1+ chronic conditions) are 30 % in total care and 60 % in primary care more likely to have any private finance compared to healthy individuals (with no chronic conditions).

**Conclusions:**

The national accounts’ “private health spending” (39 % of total spending in 2010) is not of much use regarding equity of and accessibility to medical care by the population. The mean share of uses financed privately in 2010, a more relevant measure, is 15 % with large variation between types of care and physicians. While, as under national health insurance, richer persons contribute more into the finance of (private) medical care , and sicker persons are more likely to use it, the solidarity principle—cross subsidization from the rich to the sick, which is a fundamental principle of national health insurance systems, is clearly violated.

## Background

The size and the role of private health expenditure in systems of national (social) health insurance—such as in Israel—have recently become a burning concern in many countries [1, 3, 9]. In Israel, recently, a public committee headed by the former Minister of Health (Mrs. German), was nominated specifically to explore ways to strengthen the public health system [11].

Private health expenditure includes, by definition, all expenditures which are not financed by the (broad) government. In Israel, the share of private expenditure in national health spending reached 39 % in 2012. This is one of the highest rates in the OECD: the mean over the 34 OECD systems was 28 % (OECD, 2013). The above concern, however, applies mostly to private expenditure done by the citizens. It does not relate to expenditures made by private non-profit organizations and general hospitals (such as Hadassah Medical Organization and Hadassah Medical Centers) and private donations (both constitute about 5 % of national health spending). Of the (39–5 %=) 34 % of national health spending born by the households, 8 % amount to premiums paid to voluntary (supplemental) health insurance. While this expenditure—as is the expenditure made by private non-profit organizations and hospitals—is important from national accounting and macroeconomic perspective, its consideration in relation to the social issues mentioned above is problematic, since these premiums reflect not only expected (rather than actual) expenditure on medical care, but also risk aversion.

A more meaningful—in relation to the social issues raised above—measure of the households’ health expenditures includes only direct payments on medicines, medical materials and devices, and medical care, made by the households or paid for by voluntary insurance. In Israel, these include also dental care, mental health and nursing care, most of it is presently not covered by the national health insurance system (through the sickness funds). In 2012, these expenditures constitute (34–8 %=) 26 % of national health expenditure.

The main public concern about the share of private finance in total care expenditure, has been related to inequality—the rich have higher access to private care, and will consume more of it. National health insurance systems are based on the principles of equality, fairness and solidarity—where the rich subsidize the sick. These principles imply a separation between (centralized) income-related finance and (decentralized) health needs-based delivery of service. In private health expenditure made by the households (possibly partially covered by voluntary insurance), by definition, this separation no longer holds—health services are purchased directly and individually—raising concerns about erosion in the three principles mentioned above.

In this paper, we focus on the share of private spending in total spending on medical care. Ideally, we would like to have a “finance-of-use” measure, namely, the value of total households’ finance of services—financed out of pocket or through insurance claims—in the total value of services used—financed privately or publicly. Interestingly, such a measure was introduced by the OECD in 2012 for 23 countries (see the Discussion section) but is not available for Israel. We focus instead on the share of uses having private finance in all uses of medical care.

We reiterate that we focus on health services which are included in the national insurance package of benefits. Thus, uses of dental, mental or nursing care are excluded.

Beyond calculating the above measure for different types of medical care, the analysis tries to identify the effects of income and health on the likelihood of having uses of medical care financed privately. Our interest lies in testing two empirical principles. The first, equity in finance, specifies that holding health status constant, richer persons have a higher level of expenditures, namely, finance of care. The second, vertical equity in use, specifies that sicker persons use more services.

## Methods

The data we used were taken from the Central Bureau of Statistics, which linked information on households from two surveys. The first (health) survey, which was conducted in 2009, gathered general information regarding the households and individuals and in addition gathered individuals’ health information. The second (incomes) survey, carried out in 2010, gathered both individual and household information regarding incomes. Of the 8713 households (28,968 individuals) who responded to the health survey, 7175 (24,595)—aver 80 %—were matched with information from the income survey. The main reasons for non-match were non-response and the dynamics of households’ formation: some individuals changed households over the year—due to marriage, separation etc.—and the match had no comparative meaning. Naturally, the rate of non-match is higher in the 20–40 age group.

As was mentioned above, ideally, we would like to have the amounts of money spent out of pocket or claimed from voluntary insurance for the purchase of medical care and goods. The aggregate spending would indicate how much Israelis spend privately on medical care. The share of private expenditure on medical care would be calculated as the aggregate private spending divided by the total expenditure (received by the providers of care) on medical care, publicly and privately financed. Such data is not available in Israel, let alone on an individual basis. Instead, we focused on the share of *uses* in which medical care (within the national package of benefits) was not covered totally by the national health insurance sources (through the sickness funds).

Copayments could not be identified in the data, and were disregarded.

The medical care covered by the health survey included (planned) surgeries, hospitalizations (during the previous year), doctor visits (during the previous two weeks) for each type of specialty and visits to physical therapists, speech therapists, dieticians and occupational therapists (during the previous two weeks). Note that dental, mental and nursing care are excluded. For each of these medical services we had information whether each individual had used that service in the recall period, and if so, how the service was paid for (for surgeries—how the planned surgery was to be paid for). Although the respondents were not asked specifically on the provision (purchase) of medical goods, we cannot rule out the possibility that they considered them as part of the medical care use, in particular as part of the paramedical care.

We recoded the source of payment for each use into a binary indicator: public (sickness funds within the national health insurance) vs. all other sources, namely, where any private finance is positive. Our measure of the share of private finance is the share of uses of each service, which were not solely financed by the sickness funds within the national health insurance scheme.

In addition to calculating these shares by specialties, we calculated them for all individuals who visited primary care doctors (family, internal, pediatricians and gynecologists), secondary doctors (non-primary) and all doctors. We similarly combined the paramedical encounters and all medical uses. Thus, a person can appear more than once in the list of uses, and the different appearances can have either the same or different forms of payment. The focal measure is, thus, the share of *uses* with private finance in all *uses*.

We then related the probability of having uses financed privately to the personal characteristics of the respondents: income (family income per standardized adult); health state (suffering of any of ten chronic conditions requiring a permanent use of medicines); years of schooling (0–8, 9–12, 13+); age (0–24, 25–64, 65+) and gender, both in bivariate and multivariate analyzes.

In the multivariate analysis, since we focus on uses rather than users, the observations (uses of the same user) might not be independent. In order to account for this structure, we used random-effect logistic regressions.

We note that the amount spent out of pocket on medical care could be retrieved from the CBS’ Household Expenditures Survey. This survey, however, does not include information on health and on health services utilization.

## Results

The sample consists of 43 % aged 0–24, 47 %-25–64, and 9 % over 65. Half of the respondents are women. Seventy-nine percent have no chronic conditions. Forty-nine percent have 13+ years of schooling, and mean monthly net income is IS 4131.

Table 1 indicates that in 15 % of all uses of medical care covered by the national package of services, individuals financed the care privately (i.e., in 85 % of the uses, the entire care was financed by the national health insurance system). This rate varies somewhat among the types of care studied. The lowest rate of private finance is found in primary care (10 %), followed by inpatient care (15 %) and secondary care (16 %). Uses of paramedical care have the highest private finance—in 31 % of these uses some private finance was involved.

**Table 1:** Private finance of medical care

Medical service	n of (valid) uses	% (any) private
Primary care^a^	4167	10
Secondary care^b^	1639	16
Total physicians	5806	12
Paramedical care^c^	514	31
Hospitalization^d^	1738	15
Total care	8058	15

^a^Includes family, OB/GYN, pediatrics and internal physicians

^b^Includes all other specialties

^c^Includes physical therapy, speech clinicians, dieticians, occupational therapy

^d^Including surgeries

According to Table 2, the rate of privately financed uses varies considerably along the specialty of the physician. We note that in several cases the number of uses is rather small. The under-10 %-private finance group includes pediatricians, oncologists and family physicians. The 10–15 %-private finance group includes cardiologists, ophthalmologists, skin-sex, orthopedic and ENT doctors. The 15–20 %-private finance group includes internists, OB/GYN, surgeons, endocrinologists, gastro, urologists and neurologists. The share of any-private uses among psychiatrists, pulmonologists and “other” (not appearing in the list above) doctors is above 20 %.

**Table 2:** Private finance of primary and secondary care by specialty

Specialty	n of (valid) uses	% (any) private
Pulmonologist	36	23
Psychiatrist	44	21
Neurologist	84	19
Urologist	76	19
Gastro	60	18
Endocrinologist	44	18
Surgeon	125	18
OB/GYN	253	18
Internist	61	18
ENT	147	15
Orthopedic	377	15
Skin-sex	171	14
Ophthalmologist	192	13
Cardiologist	117	11
Family physician	3078	10
Oncologist	37	9
Pediatrics	775	7
Other doctor	129	28

From Table 3 we learn that in occupational and speech therapy uses, the rate of any-private finance is high—69 and 51 % respectively. In physical therapy the rate is 26 and 15 % of the visits to dieticians are associated with any private finance.

**Table 3:** Private finance of paramedical care

Medical service	n of uses	% (any) private
Occup. therapy	25	69
Speech clinics	62	51
Physical therapy	405	26
Dieticians	22	15

Table 4 proves, in bivariate analyses, that richer individuals are more likely to finance privately medical care. The rate of uses with any private finance in the top (5th) quintile of households is double that in the poorest (1st) quintile. In paramedical care, the rate in the top quintile is 3 times the rate in the lowest quintile. A similar effect is found with relation to level of education: individuals with 13+ years of schooling are about 50 % more likely to have any private finance in total, primary care and inpatient care compared to individuals with up to 8 years of schooling. The difference is almost 100 % in paramedical care. In secondary care, the variation of the private finance rate with respect to income and education is insignificant.

**Table 4:** The population percentage spending on any private finance by selected characteristics (%)

Income quintiles	1	2	3	4	5	p
Total care	10	12	14	15	23	0.000
Hospitalization	10	15	14	16	25	0.000
Primary care	8	9	10	11	16	0.000
Secondary care	12	15	16	15	22	0.101
Paramedical care	15	24	30	34	45	0.000
Number of chronic conditions	0	1+	p			
Total care	13	14	0.248			
Hospitalization	17	14	0.099			
Primary care	9	11	0.073			
Secondary care	16	16	0.890			
Paramedical care	30	32	0.664			
Yrs. of schooling	0–8	9–12	13+	p		
Total care	10	15	16	0.000		
Hospitalization	9	17	17	0.003		
Primary care	8	10	11	0.078		
Secondary care	16	16	17	0.192		
Paramedical care	20	23	38	0.004		
Age	0–24	25–64	65–75	75+	p	
Total care	15	15	13	13	0.098	
Hospitalization	15	17	13	13	0.305	
Primary care	9	11	9	11	0.327	
Secondary care	22	15	15	14	0.029	
Paramedical care	43	26	26	23	0.002	
Gender	Men	Women	p			
Total care	15	16	0.000			
Hospitalization	15	15	0.896			
Primary care	9	11	0.047			
Secondary care	18	15	0.091			
Paramedical care	31	30	0.740			

The number of chronic conditions (0 vs. 1+) is marginally related in a bivariate analysis to the rate of any private finance in hospitalizations and primary care. However, advanced age is associated with a decreased rate of any private finance, at least in secondary and paramedical care. Women are more likely to have any private finance in total care and primary care.

Table 5 presents the odds-ratios of the explanatory variables mentioned above in multivariate random effect logistic regressions by type of care. Income clearly has remained a significant predictor of the probability to have private finance. On average, an increase of 1000 IS in net income per standardized adult increases the odds of any private finance by 10–12 % in total and primary care, by 16 % in secondary care, by 29 % in paramedical care, and by 9 % in inpatient care. Clearly, private finance of paramedical care is the most sensitive to the level of income, and of inpatient care is the least.

**Table 5:** The determinants of any private finance of medical care use (random effects logit regression)

	Any use			Primary care			Secondary care			Paramedical care			Hospitalization		
	Odds ratio	[95 % conf. interval]		Odds ratio	[95 % conf. interval]		Odds ratio	[95 % conf. interval]		Odds ratio	[95 % conf. interval]		Odds ratio	[95 % conf. interval]	
Income	**1.000**	1.000	1.000	**1.000**	1.000	1.000	**1.000**	1.000	1.000	**1.000**	1.000	1.001	**1.000**	1.000	1.000
Sickness															
0	1.000			1.000			1.000			1.000			1.000		
1+	**1.299**	1.053	1.604	**1.592**	1.079	2.350	1.773	0.872	3.605	2.296	0.768	6.864	0.777	0.543	1.114
Education															
0–8	1.000			1.000			1.000			1.000			1.000		
9–12	**1.511**	1.116	2.047	1.602	0.952	2.694	0.551	0.193	1.569	0.864	0.152	4.895	**1.772**	1.108	2.834
13+	**1.478**	1.083	2.017	1.373	0.816	2.312	0.649	0.229	1.837	1.827	0.305	10.955	1.560	0.952	2.558
Region															
Jerusalem	1.000			1.000			1.000			1.000			1.000		
North	1.091	0.640	1.859	0.675	0.268	1.700	0.357	0.058	2.203	1.755	0.173	17.817	1.750	0.639	4.796
Haifa	1.448	0.904	2.319	1.476	0.663	3.286	0.492	0.109	2.222	4.203	0.454	38.937	2.355	0.923	6.013
Center	0.979	0.701	1.367	0.906	0.528	1.555	0.589	0.201	1.729	0.733	0.170	3.153	**2.446**	1.137	5.260
Tel aviv	1.104	0.778	1.567	1.013	0.573	1.792	0.430	0.136	1.358	0.894	0.203	3.925	**3.331**	1.540	7.207
South	0.999	0.639	1.563	0.992	0.476	2.068	0.295	0.066	1.319	1.583	0.233	10.740	2.094	0.841	5.212
Judea-Samaria	1.075	0.551	2.097	2.248	0.800	6.316	0.244	0.022	2.706	18.23	0.049	40.340	0.542	0.106	2.778
Peripheral status															
Periphery	1.000			1.000			1.000			1.000			1.000		
Intermediate	1.035	0.714	1.502	0.735	0.378	1.428	1.112	0.287	4.303	1.090	0.188	6.307	1.591	0.894	2.832
Center	1.141	0.689	1.890	0.987	0.421	2.312	0.869	0.150	5.020	2.462	0.231	26.294	0.849	0.348	2.068
Age															
0–24	1.000			1.000			1.000			1.000			1.000		
25–64	0.856	0.682	1.074	1.042	0.712	1.523	**0.296**	0.129	0.678	0.122	0.020	0.751	1.086	0.724	1.630
65–74	0.712	0.501	1.013	0.817	0.453	1.474	**0.223**	0.066	0.755	0.089	0.008	1.035	1.089	0.608	1.951
75+	**0.662**	0.459	0.956	0.843	0.460	1.545	**0.158**	0.043	0.583	0.080	0.006	1.041	0.919	0.503	1.681
Gender															
Men	1.000			1.000			1.000			1.000			1.000		
Women	1.122	0.939	1.342	**1.593**	1.131	2.245	0.640	0.348	1.177	1.220	0.538	2.767	1.062	0.789	1.429
Constant	0.030	0.015	0.060	0.013	0.002	0.080	0.117	0.012	1.160	0.044	0.001	2.102	0.038	0.012	0.122
Pseudo R2		0.019			0.022			0.029			0.101			0.046	

**Bold** = O.R is significantly different from1

In the multivariate analysis, health status has a significant effect on the likelihood of having privately-financed uses in total and primary care. Those with any (1+) chronic conditions are 30 and 60 % more likely to have any private finance than those with no chronic conditions in total and primary care respectively. The private finance of secondary, inpatient and paramedical care is not related to chronic health, controlling for income.

The level of education exercises a significant effect only in total care—those with 9+ years of education are about 50 % more likely to have any private finance compared to persons with less years of schooling.

Advanced age is related to less use of any private finance in all but primary and inpatient care. In total care, persons aged 75+ are about 34 % less likely compared to persons in age group 0–24 to have any private finance. In secondary care the age gradient is clear—persons aged 25–64 are 70 %, persons aged 65–74 are 78 %, and persons aged 75+ are 84 % less likely to have any private finance compared to persons aged 0–24. In paramedical care those aged 25–64 are 88 % less likely than persons aged 0–24 to have any private finance.

Finally, controlling for all other covariates, only in primary care, women are 59 % more likely to have any private finance. Having any private finance of inpatient care in the Tel Aviv/Central district is 2.4–3.3 times more likely than in the rest of the country.

## Discussion

According to OECD definitions, 39 % of the Israeli national health spending was financed privately in 2012, one of the highest rate in the OECD. The public concern is about the deviation of the private finance from the principle underlying the national health insurance scheme enacted in 1995: from everyone according to his/her ability (to pay), to everyone according to his/her (health) needs. Such a separation can be achieved only with public finance.

We found that 15 % of the uses of any care covered by the national health insurance package of benefits (and covered in the study) were financed privately. The OECD provides international data, which is comparable, in fact, to the “ideal” finance-of-use measure mentioned earlier. From the Table “Individual health care services and medical goods” by “financing agent” in OECD.Stat, the share of private (non broad government) finance in total cost of use of medical care can be calculated for 23 countries in 2012 (for 11 countries, including Israel, data are missing). The average share is 26 % (the median is 25 %). The minimal share is found in the Netherlands (13 %), the maximal—in the US (53 %). France has a share of 21 %, Germany-23 %, and Denmark, Norway and Sweden-15–18 %. Private finance is more prevalent in the purchase of “medical goods”. Excluding these goods, namely, focusing on individual health care services only, the mean OECD share drops to 21 % (and the median—to 18 %). The minimal share is found in the Netherlands (10 %), the maximal—in the US (50 %). France has a share of 15 %, Germany-21 %, and Denmark, Norway and Sweden-11–13 %. We might conclude that the share of private finance in total cost of care in Israel is below the OECD mean. Recall, however, that dental, nursing and mental health care—where private finance is high in Israel—are not covered in this study.

The rate we found is lower (10 %) in primary care, about the same in secondary and inpatient care (15–16 %), and higher (31 %) in paramedical care, in particular in occupational (69 %) and speech (51 %) therapies. Twenty six percent of the uses of physical therapy, the most prevalent of the paramedical services, involve some private finance. The higher rate of private finance in paramedical care originates from relatively low and limited coverage by the public package of benefits.

Among physicians, family physicians and pediatricians have the lowest rate of private finance (10 and 7 % respectively). They provide the core of primary care. Interestingly, in OB/GYN, the third component of primary care, the rate of private finance is much higher–18 %, indicating the choice of many women to go private with this type of care. On the other hand, oncologists, treating life-threatening situations, have relatively low share of private finance (9 %). The specialties with the highest share of private finance are psychiatrists (21 %), pulmonologists (23 %) and “other” specialties not listed (28 %). As with paramedical care, the higher rates are related to limited coverage or availability of services.

Private finance of uses of surgeons (and probably of orthopedics and urologists)-about 20 %—are related to the choice of surgeon offered by supplementary insurance. This rate is consistent with the rate of private inpatient care (“sharap”) in Hadassah and Shearei-Tzedek medical centers, where the provision of such care is allowed.

Clearly, and as expected, controlling for health state, income has a strong positive effect, consistent across all types of care. The income effect is particularly large in paramedical care. From the equity of finance viewpoint, private finance is in accord with the principle which is at the basis of the public finance under national health insurance—rich persons pay (contribute) more than poor people. This obviously violates the principle that equally sick persons should enjoy equal care.

Controlling for income, sick persons are more likely to have any private finance in primary and total care than healthy persons are. Since in private medical care, finance equals (the value of) use (see below), we may conclude that controlling for income, sick persons are more likely to use total and primary private medical care than healthy persons are. Thus, the vertical equity principle—that sick persons receive no less medical care than healthy persons do—holds in private primary and total medical care. It does not hold in secondary, inpatient and paramedical care.

Many argue that the high rate of private finance is a result of a long and continuous effort of the Ministry of Finance to limit the public health budget, with undesired consequences for the equity of the system. Two healthcare systems—France and Australia—have intentionally and explicitly done what the Israeli government has apparently done implicitly: they encouraged the use of private insurance in order to shift the burden off the public purse. In France, with a long tradition of concern for equity, voluntary private insurance was introduced in order to cover copayments, which have increased due to freezing real public finance. Seventy nine percent of the cost of care are financed publicly (social security, AMO) [4, 8]. France is unique in that the voluntary insurance (ACS) is complementary (rather than mainly supplementary). This means that it covers essential services such as hospitalization and visits to specialists. Consequently, 90 % are covered, and a third of those without coverage give up essential medical care. The French are quite proud in the equity in access to care. This is achieved, however, with strong safety nets. First, there is a complete freedom of choice of provider and no rationing of care. Second, the voluntary insurance premiums of 7 % of the (poor) population are paid by the public. Third, patients suffering from a list of chronic conditions are exempted, by their treating physician, from copayments (ADL). Apart from clear inefficiency issues which are related to the functioning of the unregulated health insurance market, most researchers agree that the equity of the system is unsustainable facing the ever increasing healthcare costs. Increasing premiums raise the affordability problem, where less and less citizens can afford the voluntary insurance. This calls for either higher public finance of the premiums for the poor and sick, and/or the enactment of managed competition among the insurers, with risk equalization and transfers between insurers in order to keep the premiums affordable for the sick. In either case, the move would be towards greater involvement of the government, implying greater risk and income solidarity.

The Australian story is quite different, but results in similar conclusions. Introduced in 1984, the Australian Medicare public system provides universal and affordable coverage for a basic package of health benefits. Because of the increasing medical care cost and the burden on the public budget, the Australian government has encouraged private health insurance (PHI) ownership, and since 1997, introduced tax exemptions to accelerate its pick-up. Ownership rate reached 45 % in 2010, and it financed about 10 % of the national health spending. As in many other systems, PHI typically serves as a supplementary insurance, offering some “extras” to those who can afford it. In particular, it offers the choice of doctor and shorter waiting times in public hospitals [7, 10]. The equity of the system, including equity of access and equity of outcomes, and in particular regarding inpatient care, has been a major issue in the Australian health care system in recent years [5, 6]. The most publicly debated issue remains waiting time for elective surgeries in public hospitals, when waiting times in private hospitals are essentially zero [2, 6]. The threat is that increasing premiums, with increasing medical care costs, will make PHI unaffordable to many, mainly the poor and the sick. The direction in which the Australian government is heading is the enactment of managed competition among the insurers, with ex-ante risk equalization and transfers between insurers in order to keep the premiums affordable for the sick. Again, we see an emerging move towards greater involvement of the government, implying greater (risk and income) solidarity.

The Public Committee for strengthening the Israeli public health system (The German Committee) submitted its recommendations in June 2014. It calls for stronger regulation of the delivery of private care, and specific changes in the voluntary health insurance market (owned by about 80 % of the population). These changes refer, first, to unbundling the supplementary insurance policy, and the creation of a product covering choice of surgeon and second opinion, which are the most commonly used services. Second, a standardization of the terms of this product across insurers, both the HMOs and the private insurers. Third, the premiums will be community ratings according to age, and a risk-equalization mechanism will be introduced. Fourth, the insurance products will be subjected to regulation, and more and clearer information will be available to the consumers.

These recommendations seem to advocate the notion that private care and insurance should be more regulated in light of equity and efficiency principles, and they are in line with those advanced in France and Australia, namely, enhancing (risk) solidarity.

## Conclusions

Focusing on the consumption of medical care, which is the bottom line of a healthcare system, we estimate that about 15 % of the uses of non-dental care are financed privately. This is not to say in any way that the issue of private finance in the Israeli national health insurance system is less severe than implied by the OECD-national accounts numbers (39 or 26 % of national health spending according to the definition of private spending). Cross subsidization from the rich to the sick, which is a fundamental principle of national health insurance systems, is naturally non-existent in private care. Consequently, the main issue regarding the relatively low public finance of health care in Israel is not only the low level of public spending and hence the limited availability of publicly financed medical services for the (sick and poor) population. It is also the finance composition of the spending, and the total lack of solidarity, which undermines the principles of the national health insurance system.

Recent Israeli data indicates that national private health expenditure has continued to grow after 2009, the year to which the present analysis refers. This is mainly due to increases in voluntary insurance premiums and coverage. In order to identify the effect of these recent increases on equity and inequality in medical care use, newer data on “finance of use of medical care” is needed. We may speculate, however, that the share of private finance of use of services covered by the national package of benefits has continued to increase as well.
